# A systematic review of mental health interventions to reduce self-stigma in medical students and doctors

**DOI:** 10.3389/fmed.2023.1204274

**Published:** 2023-06-15

**Authors:** Amy Jean Bannatyne, Cindy Jones, Belinda M. Craig, Dominique Jones, Kirsty Forrest

**Affiliations:** ^1^Medical Program, Faculty of Health Sciences and Medicine, Bond University, Gold Coast, QLD, Australia; ^2^Menzies Health Institute Queensland, Griffith University, Gold Coast, QLD, Australia; ^3^Institute for Evidence-Based Healthcare, Faculty of Health Sciences and Medicine, Bond University, Gold Coast, QLD, Australia; ^4^Gold Coast Health and Hospital Service, Gold Coast, QLD, Australia

**Keywords:** self-stigma, doctors, medical students, stigma reduction, wellbeing, mental health, systematic review

## Abstract

**Background:**

A growing body of literature has revealed that many medical students and doctors do not seek professional help for their mental health due to fear of stigma (both public- and self-stigma) and questioning of their clinical competency. The aim of this systematic review was to identify and evaluate direct and indirect interventions that address mental health stigma in medical students and/or doctors. We focused explicitly on studies that measured the impact on self-stigma outcomes.

**Method:**

A systematic search of the following electronic databases was undertaken from inception to 13 July 2022: PubMed, Embase, PsycINFO, and CINAHL, together with manual searching of reference lists. Screening of titles, abstracts, and full texts of eligible studies, plus quality appraisal using the Mixed Methods Appraisal Tool, were independently conducted by multiple reviewers with disagreements resolved *via* discussion.

**Results:**

From 4,018 citations, five publications met the inclusion criteria. None of the studies explicitly aimed to reduce self-stigmatisation, with the majority focusing on medical students. Most of the identified interventions focused on reducing professional stigma (i.e., stigma toward patients with mental illness) and measurement of self-stigma was incidentally collected via a subscale of the general stigma measure selected. Three studies found significant reductions in self-stigma following the delivered intervention. These studies were of moderate quality, had medical student samples, employed combined education and contact interventions, and used the same outcome measure.

**Discussion:**

Intentional development and evaluation of interventions specifically designed to decrease self-stigma among doctors and medical students are needed, with further research required on the optimal components, format, length, and delivery of such interventions. Researchers delivering public/professional stigma reduction interventions should strongly consider measuring the impact of such interventions on self-stigma outcomes, using fit-for-purpose, psychometrically sound instruments.

## Introduction

The path to a career in medicine is far from easy; it is an extended period of training that requires commitment from an early age, persistence, hard work, and significant investment both emotionally and financially (1). There is no doubt the stakes are high for individuals who pursue the journey to becoming a doctor. Much has been written about the various stressors medical students and doctors encounter during their training including heavy and unpredictable workloads (often in environments where resources are stretched), sleep deprivation, pressure to excel, high stakes assessments, fear of making a mistake, and the emotional impact of human suffering and death (2, 3).

Given the pervasive stressors involved in medical training, it is not surprising that medical students and doctors are at increased risk of psychological distress and mental health conditions, relative to the general population (4). Existing literature consistently recognizes the high prevalence of anxiety (5), depressive disorders and suicidal ideation (6, 7), substance use disorders (7–9), as well as stress and burnout (10–12) in this population. Suggested solutions to improve the wellbeing of medical students and doctors have included both system level changes (e.g., modifying shift schedules, workload reductions) and individual interventions from either a treatment or prevention perspective.

While the efficacy of individual interventions is promising (12–14), participation and uptake has often been impacted by the reluctance of medical students and doctors (1). A growing body of literature has revealed that many medical students and doctors do not seek professional help for their mental health. Medical students have been found in various studies to actively avoid help-seeking and treatment (11, 15), are unwilling to disclose mental health concerns in an educational setting (16), and prefer to access support informally *via* friends, family, and sometimes peers (17). Similar observations have also been noted in doctors who frequently conceal mental health concerns from those in their professional environment and delay or fail to seek treatment (18–20). The potential consequences of avoidance in treatment seeking are two-fold. First, mental ill health can significantly impact the quality of life of medical students and doctors experiencing such symptoms. Second, mental ill health of doctors and medical students is often interconnected to the quality of care delivered (21–23).

Medical students and doctors have been found in multiple studies to report ingrained stigma toward mental illness as one of the main barriers to disclosure and help-seeking, facilitated by a belief culture that emphasizes selflessness and invincibility (24–26). Stigma is a social process of exclusion, blame, rejection, or devaluation based on a person’s characteristics or group memberships (27), which can result from the experience or anticipation of adverse social judgment. It involves three components: stereotypes (negative beliefs about a group of people/self), prejudice (negative affective response), and discrimination (behavioral response to prejudice). The ubiquitous culture of stigma within medicine is experienced in two main forms: public stigma and self-stigma (22).

Public stigma refers to negative attitudes and discriminatory behaviors carried out by others in the social environment (28). In the context of medicine, this is often expressed as negative consequences (actual or anticipated) to medical students and doctors respective academic future and career progression, damage to professional relationships, negative attitudes toward colleagues/peers experiencing mental illness (e.g., perception of “incompetence,” “weakness,” and “unreliable”), and concerns regarding confidentiality and mandatory reporting requirements as result of disclosure and treatment (22, 26, 29–35). Public stigma toward mental illness is also exhibited by medical students and doctors toward patients experiencing mental illness [known as professional stigma (36)].

Self-stigma occurs when an individual experiencing mental illness internalizes public stigma, resulting in considerable shame (28). Modified Labelling Theory (37) proposes that negative stereotypes, prejudices, and discriminatory behavior toward mental illness in an individual’s environment (i.e., workplace or university) can take on new and personal significance when the individual experiences mental ill-health. Subsequently, the individual may expect to be socially devalued by those around them (e.g., colleagues and patients), potentially leading to social withdrawal, secrecy, treatment delays/avoidance, and disempowerment which has negative implications for self-esteem, self-efficacy, and quality of life, and has also been linked to risk of suicide (38). In the context of medicine, self-stigma is often expressed as medical students or doctors experiencing significant shame and embarrassment regarding their mental health, a perception they are “weak” or a “failure,” and reduced self-esteem and self-efficacy resulting in reappraisal of their view of the world and their place within it. They also have a tendency to blame themselves for their condition and/or circumstances, thereby experiencing social withdrawal, reduced academic and/or clinical performance, avoiding or declining career opportunities, and failing to seek treatment for psychological and/or physical conditions (22, 26, 33, 39). This highlights the importance and need to address self-stigmatisation in medical students and doctors given the potential adverse impact on their health and well-being as well clinical care practices.

Given stigma is one of the main barriers to treatment seeking by both medical students and doctors, and the potential consequences of delayed treatment seeking can be significant for them and their patients, it is vital to evaluate the effectiveness of interventions aimed at reducing mental health stigma in this population. As such, the aim of this systematic review was to identify and evaluate direct and indirect interventions that address mental health stigma in medical students and/or doctors. We focused explicitly on studies that measured the impact on self-stigma outcomes as many intervention studies in this area focus on the impact to professional stigma (e.g., studies measuring stigma toward patients with conditions such as depression or schizophrenia, a form of public stigma). While improvements in public/professional stigma are positive for patients, intervention effects cannot be generalized to attitude changes experienced by medical students and doctors about their own mental health. Additionally, it is possible for medical students and doctors to hold accepting beliefs about mental illness in others (e.g., patients or colleagues), but stigmatize this vulnerability in themselves which may further constrain help-seeking (26, 39).

## Methods

The Preferred Reporting Items for Systematic Reviews and Meta-Analyses (PRISMA) (40) statement was used as a methodological framework. The review was registered on the International Prospective Register of Systematic Reviews (CRD42022312928). The data were managed and stored using Covidence (Veritas Health Innovation), an electronic systematic review platform.

### Data search

A systematic search of the following electronic databases was undertaken from inception to 13 July 2022: PubMed (Ovid), Embase (Elsevier), PsycINFO (Ovid), and CINAHL (EBSCO). With assistance from the Faculty Librarian, the search terms used included synonyms and derivatives of ‘medical professionals’ and ‘medical students’, ‘mental health’, ‘stigma’, and ‘interventions’. Complete details of the search strategy can be found in Supplementary File 1. The reference lists of the included studies were also crossed checked and relevant citations were manually searched and entered if they met the eligibility criteria.

### Eligibility criteria

Studies were included if they: (1) were primary full text articles published in English or an English translation was available; (2) the sample or subsample were medical students and/or doctors; (3) an intervention that directly or indirectly addressed mental health stigma was delivered; and (4) an outcome measuring self-stigma was reported at both pre-intervention and post-intervention. In instances where participants in a study were “health professionals,” the study was only included if data for medical students and/or doctors was reported separately. Similarly, studies where the outcome measure/s assessed various forms of stigma were only included if a subscale measuring self-stigma was reported separately at pre-intervention and post-intervention.

### Study selection

Four reviewers (AB, CJ, BC, and DJ) independently screened the titles and abstracts of all studies identified *via* the search strategy, followed by the full texts of relevant articles, using the eligibility criteria. Disagreements were resolved by consensus or by consulting with an author who had not been involved in the independent screening of a specific study.

### Data extraction

Three reviewers (AB, CJ, and BC) independently extracted data from included studies. Information extracted included: author; year of publication; country where study occurred; study aim; study design; sample characteristics; description of intervention and comparison groups; data collection (e.g., outcome measure and administration time points); main findings; and quality assessment. Disagreements were resolved by consensus.

### Data quality

The quality of included studies was assessed using the Mixed Methods Appraisal Tool (MMAT) (41), Version 18. The MMAT allows for evaluation of qualitative studies, randomized controlled trials, non-randomized studies, quantitative descriptive studies, and mixed-method studies. Rather than providing an overall quality appraisal score for each study, the MMAT recommends that pertinent details of each criterion are reported to inform the quality of the included studies.

## Results

### Results of search strategy and study selection

**Figure 1:**
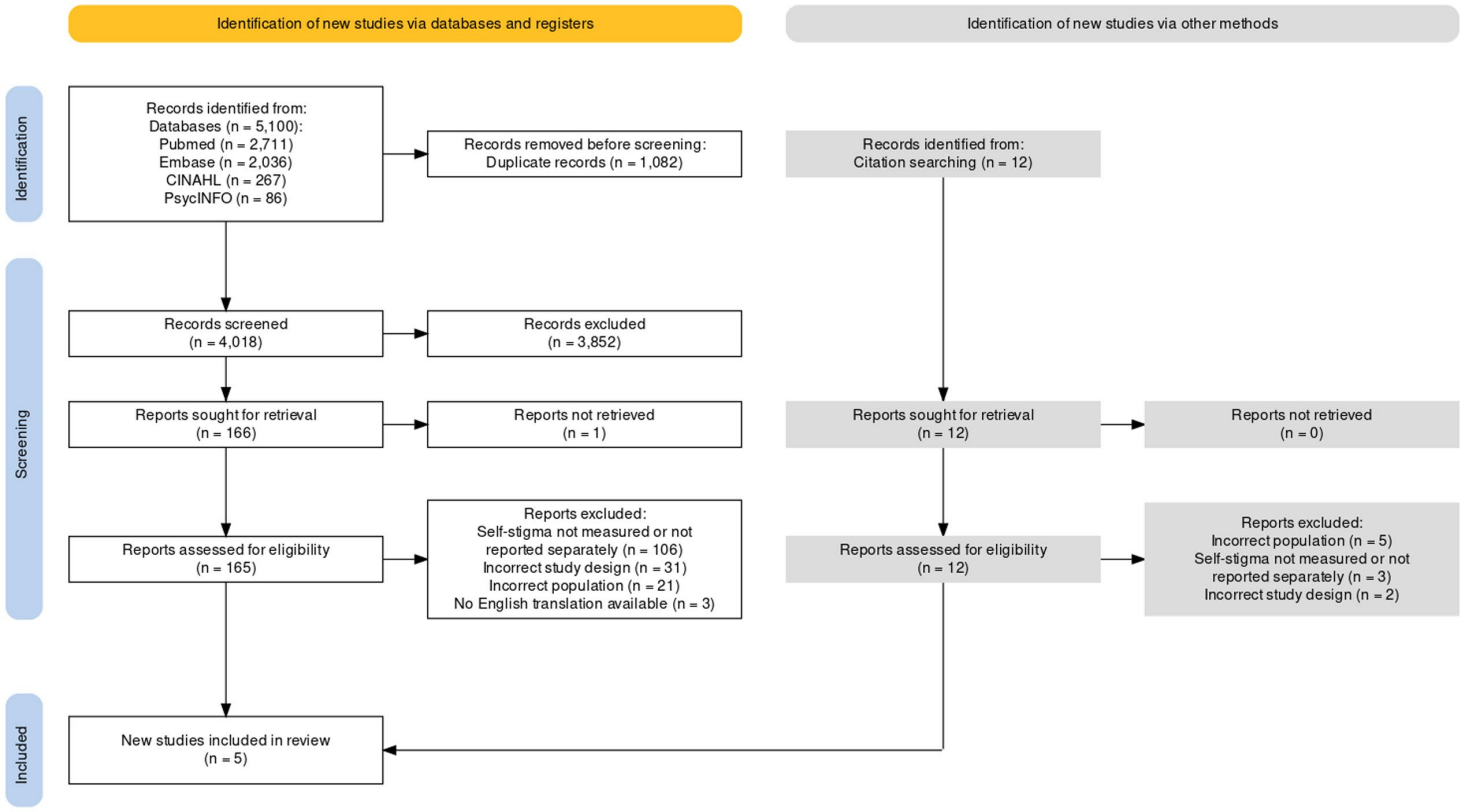
PRISMA article selection flowchart.

The literature search yielded 5,100 potentially relevant citations. After duplicates were removed, 4,018 citations were title and abstract screened, with 165 full-text articles assessed for eligibility. After full-text assessment, three citations met criteria for inclusion. Backwards citation searching from the three included studies revealed an additional 12 citations for consideration, with two additional citations meeting criteria for inclusion. This resulted in a total of five studies for inclusion in the review. The heterogeneity (e.g., study design and intervention) of the small number of studies identified precluded any scope for meta-analysis in this review, therefore a narrative synthesis was undertaken. Figure 1 depicts the PRISMA flowchart of the article selection process.

### Characteristics of included studies

Table 1 presents an overview of the included studies. Studies included in this review were conducted in Canada (42, 43), New Zealand (44), Malaysia (45), and Israel (46). The date range was 2013 to 2021. Sample sizes ranged from 49 to 243, with a total of 527 participants across all five studies. Four studies had medical student samples, two undergraduate entry (43, 45) and two postgraduate entry (44, 46) programs. One study had a sample of family physicians (42). Ages ranged from 20 to 69 across the four studies. For the four studies that reported gender information (42, 43, 45, 46), more than half of the total participants were female (62.3%).

**Table 1:** Overview of included studies.

Citation and country	Aim	Design	Sample	Intervention group	Comparison group	Data collection	Main findings	Quality assessment
Beaulieu et al. (42) Canada	Evaluate the impact of a novel skill-based approach on primary care providers’ stigma and confidence in providing care for patients with mental illness	Cluster RCT	Family physicians (*N* = 72)	(*n* = 39) 15-week intervention consisting of 3 × 3.5-h interactive workshops (contact-based education approach) and 2 × 6–8 week implementation action periods Demographics: 53.8% female /46.2% male 20.5% aged 30–39 25.6% aged 40–49 41.0% aged 50–59 12.8% aged 60–69	(*n* = 34) Wait-list control – no intervention delivered Demographics: 61.8% female/38.2% male 6.0% aged 20–29 14.7% aged 30–39 41.1% aged 40–49 29.4% aged 50–59 8.8% aged 60–69	OMS-HC (Disclosure/Help Seeking Subscale) Average score used Administered pre-intervention and post-intervention	Intervention group: Pre (*M* = 2.65, *SD* = 0.66) Post (*M* = 2.57, *SD* = 0.75) Control group: Pre (*M* = 2.63, *SD* = 0.61) Post (*M* = 2.65, *SD* = 0.52) Differences between time points were not statistically significant (*p* = 0.750)	111 family physicians initially randomized 30% incomplete data for intervention group 38% incomplete data for control group No information about adherence to intervention
Fernandez et al. (45) Malaysia	Compare the effectiveness of two different brief contact and psychoeducation-based interventions on reducing stigma in pre-clinical medical students	RCT	2nd year medical students (*N* = 102)	(*n* = 51) 90-min educational lecture +45-min face-to-face contact intervention Demographics: 72.5% female / 27.5% male Mean age of 21.10 years (*SD* = 0.30)	(*n* = 51) 90-min educational lecture +40-min video-based contact intervention Demographics: 84.3% female / 15.7% male Mean age of 21.02 years (*SD* = 0.51)	OMS-HC (Disclosure/Help Seeking Subscale) Total score used Administered pre-intervention, post-intervention and 1 month follow-up	F2F contact group: Pre (*M* = 12.58, *SD* = 0.37) Post (*M* = 9.54, *SD* = 0.40) Follow-up (*M* = 11.78, *SD* = 0.39) Video contact group: Pre (*M* = 11.94, *SD* = 0.37) Post (*M* = 9.54, *SD* = 0.40) Follow-up (*M* = 11.65, *SD* = 0.40) Authors descriptively report significant change in scores across time for both groups, but *p*-values not provided to determine between which time points and conditions.	No drop-out reported No information about adherence to intervention
Jarvie et al. (43) Canada	Determine whether a novel intervention could reduce medical students’ stigmatizing attitudes toward individuals with mental illness	Quasi experimental (no control)	2nd year medical students (*N* = 49)	(*n* = 49) 90-min large group presentation which incorporated stand-up comedy and scenes from Cracking Up (award winning documentary about Stand-Up for Mental Health [SMH]) plus Interaction with a SMH comedian in small group tutorials Demographics: 55.1% female /44.9% male 93.9% aged 20–30	No comparison or control	OMS-HC (Disclosure/Help Seeking Subscale) Average score used Administered pre-intervention and post-intervention	Intervention: Pre (*M* = 3.05, *SD* = 0.56) Post (*M* = 3.01, *SD* = 0.66) Difference between time points was not statistically significant (*p* = 0.510)	Small sample size (only 49 participants out of 130 eligible students) – impact of motivation needs to be considered No drop out reported
Newton-Howes et al. (44) New Zealand	Investigate the impact of a service user-led anti-stigma and discrimination education programme on medical student attitudes	Comparative cohort study	5th and 6th year medical students across two campuses (*N* = 243)	(*n* = 125) World of Difference Program (6-h workshop, 1-day placement with service user-led recovery-focused service, recommended readings, optional tutorials, and reflective assessments) + standard psychiatric attachment (5 weeks) Intervention was delivered to 5th year (*n* = 70) and 6th year (*n* = 55) students at Wellington campus No demographic information reported	(*n* = 118) Control – standard psychiatric attachment (5 weeks) No intervention was delivered to 5th year (*n* = 67) and 6th year (*n* = 51) students at Christchurch campus No demographic information reported	OMS-HC (Disclosure/Help Seeking Subscale) Total score used Administered at beginning of psychological medicine attachment (pre-intervention) and end of attachment (post-intervention)	Year 5 intervention group: Pre (median = 11, IQR = 10–13) Post (median = 10, IQR = 9–12) Statistically significant change (*p* = 0.003) Year 6 intervention group: Pre (median = 11, IQR = 9–12) Post (media*n* = 10, IQR = 8–12) Statistically significant change (*p* = < 0.001) Year 5 control group: Pre (median = 11, IQR = 9–13) Post (median = 10, IQR = 9–13) No statistically significant change (*p* = 0.079) Year 6 control group: Pre (median = 10, IQR = 9–12) Post (median = 10, IQR = 9–12) No statistically significant change (*p* = 0.075)	Eligibility criteria unclear Unclear how many participants across each year in each campus were eligible Cannot determine whether sample was representative of population Unclear whether confounding variables were controlled for No information about adherence to intervention
Martin et al. (46) Israel	Assess the impacts of physicians’ sharing their lived experiences of overcoming serious life challenges as an educational intervention to combat mental health stigma (self-stigma).	Mixed methods	2nd year medical students (*N* = 61)	(*n* = 61) Contact-educational intervention consisting of three components: (1) lived experience sharing by 3 senior physicians; (2) small group debriefing with 1 of the 3 senior physicians; (3) educational materials on mental health available to students. Demographics: 45.9% female / 54.1% male 65.6% aged 25 to 29 34.4% aged 24 and under	No comparison or control	OMS-HC (Disclosure/Help Seeking Subscale) Total score used Administered pre-intervention and post-intervention	Intervention: Pre (*M* = 14.40, *SD* = 3.10) Post (*M* = 12.90, *SD* = 3.30) Difference between time points was statistically significant (*p* = < 0.001) *Analysis based on 53 students.	61 students invited, but only 53 completed T1 and T2 (12.7% dropout). Small sample size and unclear if participants are representative of target population. Unclear whether confounding variables were controlled for

All five studies explored the impact of combined educational and contact-based interventions on stigma outcomes. Two studies were randomized controlled designs with either a wait-list control (42) or a comparison intervention (45). One study was a comparative cohort study comparing cohorts across two campuses who undertook a standard psychiatric rotation and then either received or did not receive a stigma reduction intervention (44). Two studies were quasi-experimental design with no control (43, 46), with Martin et al. (46) also incorporating a qualitative component. Three studies delivered the intervention in a single session (43, 45, 46), while the other two studies delivered the intervention over a series of weeks with several components (42, 44). The Disclosure/Help-Seeking Subscale of the Opening Minds Scale for Health Care Providers (OMS-HC) (47) was used as a measure of self-stigma across all five studies, with administration at pre-intervention and post-intervention. Only one study (45) included a follow-up period (1 month). All five studies aimed to reduce stigma in general. None specifically focused on reducing self-stigma.

### Intervention impact on self-stigma

Results varied across studies, with three studies finding significant improvements in self-stigma. Newton-Howes et al. (44), which examined the effectiveness of the World of Difference Program in addition to a standard 5-week psychiatric attachment, found a statistically significant improvement in self-stigma from pre-intervention to post-intervention in Year 5 and Year 6 medical students who received the intervention (*p* = 0.003 and <0.001, respectively). No significant changes were found in Year 5 and Year 6 medical students who completed a psychiatric rotation but did not receive the intervention (*p* = 0.079 and 0.075, respectively).

Martin et al. (46), which examined the effectiveness of a contact-educational intervention with medicine students consisting of three components: (1) lived experience sharing by three senior physicians on topics such as failing high stakes examinations and personal experiences of mental health conditions and treatment (2) small group debriefing with one of the three senior physicians; (3) educational materials on mental health available to students, reported a statistically significant reduction in self-stigma from pre-intervention to post-intervention.

Fernandez et al. (45), which examined the effectiveness of a 90-min educational lecture combined with either a 45-min face-to-face contact intervention or a 40-min video-based contact intervention in medical students, reported that both groups experienced statistically significant reductions in self-stigma across time. However, significance values were not provided and therefore it cannot be determined which time points demonstrated statistically significant changes. Review of outcome scores in Fernandez et al. (45) indicates that both groups experienced a mean reduction of 2.40–3.00 points on the Disclosure and Help-Seeking Subscale between pre-intervention and post-intervention. A mean increase of 2.11–2.24 points was reported in both groups between post-intervention and the 1-month follow-up, with follow-up scores within 0.8 (face-to-face contact group) and 0.3 (video-based contact group) points of the pre-intervention scores. This potentially indicates that for both groups there was a reduction in self-stigma from pre-intervention to post-intervention, but the intervention effect was not maintained at follow-up.

The other two studies did not find a significant reduction in self-stigma. Beaulieu et al. (42), which examined the effectiveness of a 15-week intervention consisting of both education and contact components against a wait-list control in family physicians, found there was no significant change in average scores on the Disclosure and Help-Seeking Subscale between pre-intervention and post-intervention for either group (*p* > 0.05). Lastly, Jarvie et al. (43) examined the effectiveness of a 90-min educational presentation and interaction with Stand-Up for Mental Health comedians (who had previous experiences of mental illness) in medical students. They found no significant change in average scores on the Disclosure and Help-Seeking Subscale between pre-intervention and post-intervention (*p* = 0.510).

### Quality assessment

Overall, the quality of the five studies was poor to fair. All studies met the first two screening criteria of the MMAT. For the two RCT studies (42, 45), both described an adequate randomization process, and the groups were similar at baseline. Complete outcome data was provided in Fernandez et al. (45); however, Beaulieu et al. (42) had considerable withdrawal/dropout from randomization, ranging from 30% for the intervention group to 38% for the control group. Previous literature has indicated acceptable withdrawal/dropout rates range from 5% (48) to 20% (49), therefore the withdrawal/dropout observed in Beaulieu et al. (42) likely impacts quality. In both studies, participants were the outcome assessors and not blinded to the intervention provided. Adherence to the assigned intervention was not described in either study, though more likely to have occurred in Fernandez et al. (45) due to the brief, time-limited intervention period and implementation as a part of scheduled educational activities.

For the non-randomized studies (43–46), it was unclear if participants were representative of the target population. Only 49 of 130 eligible participants agreed to participate (response rate = 37.7%) in Jarvie et al. (43), while no eligibility criteria or number of students potentially eligible were described in Newton-Howes et al. (44) or Martin et al. (46). All three studies met criteria for appropriate outcome and intervention measures. The relatively low response rate and small sample size (*N* = 49) reported in Jarvie et al. (43) also needs to be considered in the context of participant motivation, which may have impacted findings. Incomplete data ranged from 9 to 12% for Newton-Howes et al. (44) and 12.7% for Martin et al. (46); however, this is within acceptable ranges according to the MMAT criteria. Complete data was reported for Jarvie et al. (43); however, this likely reflects the brief, time-limited intervention period. In all three studies, the interventions appear to have been administered as intended, though this is not explicitly stated. None of three studies appear to account, or control, for confounding variables in the design or analysis. A summary of quality appraisal can be found in Table 1.

## Discussion

This systematic review highlights the paucity of research examining the effectiveness of direct and indirect interventions to reduce self-stigmatisation in medical students and doctors. None of the five included studies in this review explicitly aimed to reduce self-stigmatisation, with the majority focusing on medical students. Most of the identified interventions focused on reducing professional stigma (i.e., stigma toward patients with mental illness) and measurement of self-stigma was incidentally collected in the stigma measure selected. Only three studies found significant reductions in self-stigma following the delivered intervention. These studies were of moderate quality, had medical student samples, employed combined education and contact interventions, and used the same outcome measure.

Despite the lack of explicit focus on self-stigma in both intervention design and outcome measurement, findings of this review indicate that aspects of interventions to reduce public and/or professional stigma may be effective in reducing self-stigma, as self-disclosure and help seeking intentions had positive influence in three studies of varying intervention lengths (e.g., one-off intervention vs. multiple component intervention delivered over several weeks). This is not surprising as self-stigma is strongly related and interconnected to broader structural and public forms of stigma, therefore challenging and breaking down stigmatizing attitudes and behaviors at a public and institutional level may limit individual exposure to such attitudes and behaviors, potentially preventing or minimizing internalization of stigmatizing beliefs (50). However, the impact of public and professional stigma reduction interventions on self-stigma outcomes was not consistent across all studies in this review, and there were a considerable number of studies excluded for not measuring the impact on self-stigma. This highlights the importance and need for researchers to evaluate the effectiveness of public and professional stigma reduction interventions on self-stigma outcomes, ideally using psychometrically sound instruments that focus entirely on measuring self-stigma such as the Internalized Stigma of Mental Illness scale (51) or Self Stigma of Mental Illness scale (52), rather than instruments that incidentally measure self-stigma *via* one subscale.

More importantly, however, there is a clear priority for researchers to intentionally create and evaluate the effectiveness of interventions aimed at explicitly reducing self-stigma in doctors and medical students who, as a group, have been identified at increased risk of experiencing self-stigma (50). At the current time, there appears to be almost no published research about the effectiveness of interventions aimed explicitly at reducing/preventing self-stigma in doctors and/or medical students. The small number of studies that have examined the effectiveness of interventions to reduce self-stigma have mostly involved community members with serious mental illness (e.g., schizophrenia) and utilized techniques from Cognitive Behavioral Therapy (CBT) and mindfulness, including psychoeducation to correct misunderstandings about mental illness, cognitive restructuring to challenging self-stigmatizing thoughts, approaches to strengthen personal abilities, and coping strategies for dealing with public stigma. These interventions have been delivered in individual and group settings (53–56). The main goal of these approaches is to enhance resilience, self-compassion, and the ability to withstand stigma by highlighting personal strengths and emphasizing concepts such as hope, recovery, relapse prevention, self-efficacy, and meaning (57).

While CBT and mindfulness based self-stigma approaches used in community samples may also be useful for targeting self-stigma in doctors and medical students, results of this review suggest that incorporating lived experience sharing (i.e., contact) from individuals in the workplace and/or educational environment may be a key feature of future self-stigma interventions with this population. Contact interventions put a face to stigmatized conditions and have the potential to normalize the experience of mental illness in the medical community (58), while emphasizing the benefits of help-seeking and the ability to continue providing quality patient care in many circumstances (45, 59).

At present, the optimal components, format, length, and delivery of self-stigma intervention for medical students and doctors is unknown and warrants further research *via* expert consensus methods such as a Delphi study. It is likely that an intervention (co-designed with doctors and medical students that have experienced self-stigma) that combines both education, lived experience contact from a mix of senior and junior staff members from various specialities, therapeutic elements of CBT, self-compassion exercises, and empowerment through activism to foster stigma resistance, could be an ideal starting point for evaluation (57, 60–62). Naturally, any individual system level effort needs to be combined with broader stigma-reduction interventions and policies to address both public and structural stigma and discrimination within medicine, and system changes to improve overall wellbeing for all staff.

### Limitations

It is acknowledged the results of this review are limited by the small number of studies available for inclusion with relatively small sample sizes, varying study quality, and heterogeneity. This issue highlights the dearth of research in this area. Additionally, in the five included studies, participants were likely aware of group assignment and it plausible that demand characteristics influenced results in a favorable manner, particularly for studies administered in a pre-post design with an intervention delivered by a known/liked educator [e.g., (45, 46)]. It should also be noted that only one of the five studies included in this review included follow-up [e.g., (45)], therefore it is unknown whether any impact to self-stigma from interventions addressing public/professional stigma is maintained, a well highlighted issue in stigma research (63). Future research should ensure that any evaluation of intervention effectiveness includes appropriate follow-up to determine whether intervention effects are maintained in the short- and long- term (63), ideally using a robust and psychometrically sound measure/s of self-stigma.

## Conclusion

This review highlighted a lack of research examining the effectiveness of direct and indirect interventions to reduce self-stigmatization in medical students and doctors, a group that are well known to be at increased risk of psychological distress and various mental health conditions (4, 7) and more susceptible to the internalization of stigmatizing attitudes due to the invincibility belief culture in medicine (26, 50). Intentional development and evaluation of interventions specifically designed to decrease self-stigma among doctors and medical students are needed, with further research required on the optimal components, format, length, and delivery of such interventions. Looking to self-stigma interventions developed for other populations, along with expert consensus methods such as a Delphi study, may be beneficial to clarify these knowledge gaps prior to intervention development. In the interim, researchers delivering public/professional stigma reduction interventions should strongly consider measuring the impact of such interventions on self-stigma outcomes, using fit-for-purpose, psychometrically sound instruments.

## Author contributions

AB, CJ, BC, and KF contributed substantially to the conceptualization and refinement of the idea, with DJ producing the study protocol. AB, CJ, BC, and DJ developed and refined the search string, with DJ undertaking the systematic searches. AB, CJ, BC, and DJ jointly completed the data screening, extraction, and quality appraisal. AB drafted the initial manuscript, with CJ, BC, and KF contributing with constructive feedback. All authors approved the final manuscript.

## Conflict of interest

The authors declare the research was conducted in the absence of any commercial or financial relationships that could be construed as a potential conflict of interest.

## Publisher’s note

All claims expressed in this article are solely those of the authors and do not necessarily represent those of their affiliated organizations, or those of the publisher, the editors and the reviewers. Any product that may be evaluated in this article, or claim that may be made by its manufacturer, is not guaranteed or endorsed by the publisher.

## References

[1] Grant A Kowalczuk J Marrin K Porter A Rix A. Trainee doctors’ views on mental illness among their peers and access to support services. Int Rev Psychiatry. (2019) 31:588–97. doi: 10.1080/09540261.2019.1616893, PMID: 31184532

[2] Dyrbye LN Thomas MR Shanafelt TD. Medical student distress: causes, consequences, and proposed solutions. Mayo Clin Proc. (2005) 80:1613–22. doi: 10.4065/80.12.1613 16342655

[3] Hill MR Goicochea S Merlo LJ. In their own words: stressors facing medical students in the millennial generation. Med Educ. (2018) 23:1530558. doi: 10.1080/10872981.2018.1530558 PMC6179084 30286698

[4] Wu F Ireland M Hafekost K Lawrence D. National mental health survey of doctors and medical students. (2019). Available at: https://www.beyondblue.org.au/docs/default-source/research-project-files/bl1132-report—nmhdmss-full-report_web

[5] Kroenke K Spitzer RL Williams JB Monahan PO Löwe B. Anxiety disorders in primary care: prevalence, impairment, comorbidity, and detection. Ann Intern Med. (2007) 146:317–25. doi: 10.7326/0003-4819-146-5-200703060-00004, PMID: 17339617

[6] Dyrbye LN Thomas MR Shanafelt TD. Systematic review of depression, anxiety, and other indicators of psychological distress among US and Canadian medical students. Acad Med. (2006) 81:354–73. doi: 10.1097/00001888-200604000-00009, PMID: 16565188

[7] Rotenstein LS Ramos MA Torre M Segal JB Peluso MJ Guille C. Prevalence of depression, depressive symptoms, and suicidal ideation among medical students: a systematic review and meta-analysis. JAMA. (2016) 316:2214–36. doi: 10.1001/jama.2016.17324, PMID: 27923088 PMC5613659

[8] Ghodse H Galea S. Misuse of drugs and alcohol. Understanding doctors' performance. New York, NY CRC Press. (2006) 38–47

[9] Kumar P Basu D. Substance abuse by medical students and doctors. J Indian Med Assoc. (2000) 98:447–52. PMID: 11294326

[10] Brooks SK Gerada C Chalder T. Review of literature on the mental health of doctors: are specialist services needed? J Ment Health. (2011) 20:146–56. doi: 10.3109/09638237.2010.541300, PMID: 21275504

[11] Dahlin ME Runeson B. Burnout and psychiatric morbidity among medical students entering clinical training: a three year prospective questionnaire and interview-based study. BMC Med Educ. (2007) 7:1–8. doi: 10.1186/1472-6920-7-6 17430583 PMC1857695

[12] Niemi P Vainiomäki P. Medical students’ distress–quality, continuity and gender differences during a six-year medical programme. Med Teach. (2006) 28:136–41. doi: 10.1080/01421590600607088, PMID: 16707294

[13] Petrie K Crawford J Baker ST Dean K Robinson J Veness BG. Interventions to reduce symptoms of common mental disorders and suicidal ideation in physicians: a systematic review and meta-analysis. Psychiatry. (2019) 6:225–34. doi: 10.1016/S2215-0366(18)30509-1, PMID: 30744997

[14] West CP Dyrbye LN Erwin PJ Shanafelt TD. Interventions to prevent and reduce physician burnout: a systematic review and meta-analysis. Lancet. (2016) 388:2272–81. doi: 10.1016/S0140-6736(16)31279-X, PMID: 27692469

[15] Schwenk TL Davis L Wimsatt LA. Depression, stigma, and suicidal ideation in medical students. JAMA. (2010) 304:1181–90. doi: 10.1001/jama.2010.1300, PMID: 20841531

[16] Miller S Ross S Cleland J. Medical students’ attitudes towards disability and support for disability in medicine. Med Teach. (2009) 31:e272–7. doi: 10.1080/01421590802516814, PMID: 19811160

[17] Brimstone R Thistlethwaite JE Quirk F. Behaviour of medical students in seeking mental and physical health care: exploration and comparison with psychology students. Med Educ. (2007) 41:74–83. doi: 10.1111/j.1365-2929.2006.02649.x, PMID: 17209895

[18] Cohen D Winstanley S Greene G. Understanding doctors’ attitudes towards self-disclosure of mental ill health. Occup Med. (2016) 66:383–9. doi: 10.1093/occmed/kqw024, PMID: 27030052 PMC4913366

[19] Kay M Mitchell G Clavarino A Doust J. Doctors as patients: a systematic review of doctors' health access and the barriers they experience. Br J Gen Pract. (2008) 58:501–8. doi: 10.3399/bjgp08X319486, PMID: 18611318 PMC2441513

[20] Winter P Rix A Grant A. Medical student beliefs about disclosure of mental health issues: a qualitative study. J Vet Med Educ. (2017) 44:147–56. doi: 10.3138/jvme.0615-097R, PMID: 28206830

[21] Firth-Cozens J. Interventions to improve physicians’ well-being and patient care. Soc Sci Med. (2001) 52:215–22. doi: 10.1016/S0277-9536(00)00221-5 11144777

[22] Forbes MP Iyengar S Kay M. Barriers to the psychological well-being of Australian junior doctors: a qualitative analysis. BMJ Open. (2019) 9:027558. doi: 10.1136/bmjopen-2018-027558, PMID: 31196900 PMC6575636

[23] Wallace JE Lemaire JB Ghali WA. Physician wellness: a missing quality indicator. Lancet. (2009) 374:1714–21. doi: 10.1016/S0140-6736(09)61424-0 19914516

[24] Guille C Speller H Laff R Epperson CN Sen S. Utilization and barriers to mental health services among depressed medical interns: a prospective multisite study. J Grad Med. (2010) 2:210–4. doi: 10.4300/JGME-D-09-00086.1, PMID: 21975622 PMC2941380

[25] Fox FE Doran NJ Rodham KJ Taylor GJ Harris MF O’Connor M. Junior doctors’ experiences of personal illness: a qualitative study. Med Educ. (2011) 45:1251–61. doi: 10.1111/j.1365-2923.2011.04083.x 22026816

[26] Sukhera J Poleksic J Zaheer J Pack R. Normalising disclosure or reinforcing heroism? An exploratory critical discourse analysis of mental health stigma in medical education. Med Educ. (2022) 56:823–33. doi: 10.1111/medu.14790 35246993

[27] Goffman E. Stigma: *notes on the management of spoiled identity*. New York: Simon and Schuster (2009).

[28] Corrigan PW Powell KJ Rüsch N. How does stigma affect work in people with serious mental illnesses? Psychiatr Rehabil J. (2012) 35:381–4. doi: 10.1037/h0094497, PMID: 23116379

[29] Dyrbye LN Eacker A Durning SJ Brazeau C Moutier C Massie FS. The impact of stigma and personal experiences on the help-seeking behaviors of medical students with burnout. Acad Med. (2015) 90:961–9. doi: 10.1097/ACM.0000000000000655, PMID: 25650824

[30] Ey S Moffit M Kinzie JM Choi D Girard DE. “If you build it, they will come”: attitudes of medical residents and fellows about seeking services in a resident wellness program. J Grad Med Educ. (2013) 5:486–92. doi: 10.4300/JGME-D-12-00048.1, PMID: 24404315 PMC3771181

[31] Grant A Rix A Mattick K., Winter P., Jones D. Identifying good practice among medical schools in the support of medical students with mental health concerns: A report prepared for the General Medical Council. (2013). Available at: https://www.gmc-uk.org/-/media/documents/Identifying_good_practice_among_medcal_schools_in_the_support_of_students_with_mental_health_concerns.pdf_52884825.pdf

[32] Grant A Rix A Shrewsbury D. ‘If you're crying this much you shouldn't be a consultant’: the lived experience of UK doctors in training with mental illness. Int Rev Psychiatry. (2019) 31:673–83. doi: 10.1080/09540261.2019.1586326, PMID: 31084443

[33] Henderson M Brooks SK del Busso L Chalder T Harvey SB Hotopf M. Shame! Self-stigmatisation as an obstacle to sick doctors returning to work: a qualitative study. BMJ Open. (2012) 2:e001776. doi: 10.1136/bmjopen-2012-001776, PMID: 23069770 PMC3488732

[34] Pheister M Peters RM Wrzosek MI. The impact of mental illness disclosure in applying for residency. Acad Psychiatry. (2020) 44:554–61. doi: 10.1007/s40596-020-01227-8, PMID: 32415458

[35] Shahaf-Oren B Madan I Henderson C. “A lot of medical students, their biggest fear is failing at being seen to be a functional human”: disclosure and help-seeking decisions by medical students with health problems. BMC Med Educ. (2021) 21:1–10. doi: 10.1186/s12909-021-03032-9 34865636 PMC8645095

[36] Ahmedani BK. Mental health stigma: society, individuals, and the profession. J Soc Work Values Ethics. (2011) 8:4–1. PMC3248273 22211117

[37] Link BG Cullen FT Struening E Shrout PE Dohrenwend BP. A modified labeling theory approach to mental disorders: an empirical assessment. Am Sociol Rev. (1989) 54:400–23. doi: 10.2307/2095613

[38] Dubreucq J Plasse J Franck N. Self-stigma in serious mental illness: a systematic review of frequency, correlates, and consequences. Schizophr Bull. (2021) 47:1261–87. doi: 10.1093/schbul/sbaa181, PMID: 33459793 PMC8563656

[39] Wimsatt LA Schwenk TL Sen A. Predictors of depression stigma in medical students: potential targets for prevention and education. Am J Prev Med. (2015) 49:703–14. doi: 10.1016/j.amepre.2015.03.021, PMID: 26141915

[40] Moher D Liberati A Tetzlaff J Altman DG PRISMA Group. Preferred reporting items for systematic reviews and meta-analyses: the PRISMA statement. Ann Intern Med. (2009) 151:264–9. doi: 10.7326/0003-4819-151-4-200908180-00135 19622511

[41] Hong QN Fàbregues S Bartlett G Boardman F Cargo M Dagenais P. The Mixed Methods Appraisal Tool (MMAT) version 2018 for information professionals and researchers. Educ Inf. (2018) 34:285–91. doi: 10.3233/EFI-180221

[42] Beaulieu T Patten S Knaak S Weinerman R Campbell H Lauria-Horner B. Impact of skill-based approaches in reducing stigma in primary care physicians: results from a double-blind, parallel-cluster, randomized controlled trial. Can J Psychiatr. (2017) 62:327–35. doi: 10.1177/0706743716686919, PMID: 28095259 PMC5459227

[43] Jarvie A Buxton JA Szeto ACH Austin J. A pilot study of the effect of exposure to stand-up comedy performed by individuals with mental illness on medical students’ stigmatization of those affected. UBCMJ. (2013) 5:15–8.

[44] Newton-Howes G Senior J Beaglehole B Purdie GL Gordon SE. Does a comprehensive service user-led education programme effect more positive attitudes towards recovery and less stigmatising attitudes towards people with lived experience of mental distress in medical students? A comparative cohort study. Aust N Z J Psychiatry. (2021) 55:903–10. doi: 10.1177/0004867420987886, PMID: 33459033

[45] Fernandez A Tan K-A Knaak S Chew BH Ghazali SS. Effects of brief psychoeducational program on stigma in Malaysian pre-clinical medical students: a randomized controlled trial. Acad Psychiatry. (2016) 40:905–11. doi: 10.1007/s40596-016-0592-1, PMID: 27527730

[46] Martin A Chilton J Paasche C Nabatkhorian N Gortler H Cohenmehr E. Shared living experiences by physicians have a positive impact on mental health attitudes and stigma among medical students: a mixed-methods study. J Med Educ Curric. (2020) 7:238212052096807–9. doi: 10.1177/2382120520968072 PMC7594232 33195803

[47] Kassam A Papish A Modgill G Patten S. The development and psychometric properties of a new scale to measure mental illness related stigma by health care providers: the Opening Minds Scale for Health Care Providers (OMS-HC). BMC Psychiatry. (2012) 12:1–12. doi: 10.1186/1471-244X-12-62 22694771 PMC3681304

[48] MacLehose R Reeves B Harvey I Sheldon T Russell I Black A. A systematic review of comparisons of effect sizes derived from randomised and non-randomised studies. Health Technol Assess. (2000) 4:1–154. doi: 10.3310/hta4340, PMID: 11134917

[49] Van Tulder M Furlan A Bombardier C Bouter L Editorial Board of the Cochrane Back Review Group. Updated method guidelines for systematic reviews in the Cochrane collaboration back review group. Spine. (2003) 28:1290–9. doi: 10.1097/01.BRS.0000065484.95996.AF 12811274

[50] Australian Government National Mental Health Commission. Draft national stigma and discrimination reduction strategy. (2022). Available at: https://www.mentalhealthcommission.gov.au/projects/stigma-and-discrimination-reduction-strategy (Accessed January 2023).

[51] Ritsher JB Otilingam PG Grajales M. Internalized stigma of mental illness: psychometric properties of a new measure. Psychiatry Res. (2003) 121:31–49. doi: 10.1016/j.psychres.2003.08.008, PMID: 14572622

[52] Corrigan PW Michaels PJ Vega E Gause M Watson AC Rüsch N. Self-stigma of mental illness scale—short form: reliability and validity. Psychiatry Res. (2012) 199:65–9. doi: 10.1016/j.psychres.2012.04.009, PMID: 22578819 PMC3439592

[53] Corrigan PW Calabrese JD. Strategies for assessing and diminishing self-stigma In: Corrigan PW, editor. On the stigma of mental illness: Practical strategies for research and social change. Washington, DC: American Psychological Association (2005). 239–56.

[54] Tsang HW Ching S Tang K Lam H Law PY Wan C. Therapeutic intervention for internalized stigma of severe mental illness: a systematic review and meta-analysis. Schizophr Res. (2016) 173:45–53. doi: 10.1016/j.schres.2016.02.013, PMID: 26969450

[55] Wood L Byrne R Varese F Morrison AP. Psychosocial interventions for internalised stigma in people with a schizophrenia-spectrum diagnosis: a systematic narrative synthesis and meta-analysis. Schizophr Res. (2016) 176:291–303. doi: 10.1016/j.schres.2016.05.001, PMID: 27256518

[56] Yanos PT Lucksted A Drapalski AL Roe D Lysaker P. Interventions targeting mental health self-stigma: a review and comparison. Psychiatr Rehab J. (2015) 38:171–8. doi: 10.1037/prj0000100, PMID: 25313530 PMC4395502

[57] Groot C. Understanding how to address self-stigma about mental health: a report prepared for the National Mental Health Commission to inform the National Stigma and Discrimination Reduction Strategy. (2021). Available at: https://haveyoursay.mentalhealthcommission.gov.au/72951/widgets/353888/documents/243977

[58] Brower KJ. Professional stigma of mental health issues: physicians are both the cause and solution. Acad Med. (2021) 96:635–40. doi: 10.1097/ACM.0000000000003998, PMID: 33885412 PMC8078109

[59] Smith LD Peck PL McGovern RJ. Comparison of medical students, medical school faculty, primary care physicians, and the general population on attitudes toward psychological help-seeking. Psychol Rep. (2002) 91:1268–72. doi: 10.2466/pr0.2002.91.3f.1268, PMID: 12585548

[60] Chan KKS Lee CW Mak WW. Mindfulness model of stigma resistance among individuals with psychiatric disorders. Mindfulness. (2018) 9:1433–42. doi: 10.1007/s12671-018-0887-2

[61] Firmin RL Luther L Lysaker PH Minor KS Salyers MP. Stigma resistance is positively associated with psychiatric and psychosocial outcomes: a meta-analysis. Schizophr Res. (2016) 175:118–28. doi: 10.1016/j.schres.2016.03.008, PMID: 27102423

[62] Post F Buchta M Kemmler G Pardeller S Frajo-Apor B Hofer A. Resilience predicts self-stigma and stigma resistance in stabilized patients with bipolar I disorder. Front Psych. (2021) 12:678807. doi: 10.3389/fpsyt.2021.678807, PMID: 34093288 PMC8176112

[63] Jorm AF. Effect of contact-based interventions on stigma and discrimination: a critical examination of the evidence. Psychiatr Serv. (2020) 71:735–7. doi: 10.1176/appi.ps.201900587, PMID: 32188364

