# Supplementary material for: A systematic review of mental health interventions to reduce self-stigma in medical students and doctors
Source: Front Med (Lausanne). 2023 Jun 15;10:1204274. doi: 10.3389/fmed.2023.1204274 (PMC10311217; doi:10.3389/fmed.2023.1204274)
Supplement: Supplementary file 1 [file Data_Sheet_1.docx]

| **Database** | **Search String** |
| --- | --- |
| PubMed | (doctor*[tiab] OR physician*[tiab] OR "medical practitioner*"[tiab] OR "healthcare professional*"[tiab] OR "student doctor*"[tiab] OR "medical student*"[tiab] OR "medical trainee*"[tiab] OR "med student*"[tiab] OR "general practitioner*"[tiab] OR Physicians[Mesh] OR "Students, medical"[Mesh]) **AND** ("mental health"[tiab] OR "mental illness*"[tiab] OR "mental well-being"[tiab] OR "mental wellbeing"[tiab] OR "psychosocial health"[tiab] OR "psychosocial well-being"[tiab] OR "psychosocial wellbeing"[tiab] OR Depression[tiab] OR Anxiety[tiab] OR Suicide[tiab] OR Burnout[tiab] OR "Mental health"[Mesh] OR Depression[Mesh] OR Anxiety[Mesh] OR Suicide[Mesh] OR "Burnout, Psychological"[Mesh]) **AND** (Stigma[tiab] OR attribute*[tiab] OR perception*[tiab] OR attitude*[tiab] OR "Social Stigma"[Mesh] OR Perception[Mesh]) **AND** (“educational intervention*”[tiab] OR program*[tiab] OR training*[tiab] OR course*[tiab] OR curriculum*[tiab] OR "Education"[Mesh]) |
| Embase | (doctor*:ti,ab OR physician*:ti,ab OR "medical practitioner*":ti,ab OR "healthcare professional*":ti,ab OR "student doctor*":ti,ab OR "medical student*":ti,ab OR "medical trainee*":ti,ab OR "med student*":ti,ab OR "general practitioner*":ti,ab OR 'Physician'/exp OR ‘medical student’/exp) **AND** ("mental health":ti,ab OR "mental illness*":ti,ab OR "mental well-being":ti,ab OR "mental wellbeing":ti,ab OR "psychosocial health":ti,ab OR "psychosocial well-being":ti,ab OR "psychosocial wellbeing":ti,ab OR Depression:ti,ab OR Anxiety:ti,ab OR Suicide:ti,ab OR Burnout:ti,ab OR 'mental health'/exp OR 'Depression'/exp OR 'Anxiety'/exp OR 'Suicide'/exp OR 'Burnout’/exp) **AND** (Stigma:ti,ab OR attribute*:ti,ab OR perception*:ti,ab OR attitude*:ti,ab OR 'Social Stigma'/exp OR 'perception'/exp) **AND** (educational intervention*:ti,ab OR program*:ti,ab OR training*:ti,ab OR course*:ti,ab OR curriculum*:ti,ab) |
| CINAHL | ((TI doctor* OR AB doctor*) OR (TI physician* OR AB physician*) OR (TI "medical practitioner*" OR AB "medical practitioner*") OR (TI "healthcare professional*" OR AB "healthcare professional*") OR (TI "student doctor*" OR AB "student doctor*") OR (TI "medical student*" OR AB "medical student*") OR (TI "medical trainee*" OR AB "medical trainee*") OR (TI "med student*" OR AB "med student*") OR (TI "general practitioner*" OR AB "general practitioner*") OR (MH "Physicians+") OR (MH "students, medical+")) **AND** ((TI "mental health" OR AB "mental health") OR (TI "mental illness*" OR AB "mental illness*") OR (TI "mental well-being" OR AB "mental well-being") OR (TI "mental wellbeing" OR AB "mental wellbeing") OR (TI "psychosocial health" OR AB "psychosocial health") OR (TI "psychosocial well-being" OR AB "psychosocial well-being") OR (TI "psychosocial wellbeing" OR AB "psychosocial wellbeing") OR (TI Depression OR AB Depression) OR (TI Anxiety OR AB Anxiety) OR (TI Suicide OR AB Suicide) OR (TI Burnout OR AB Burnout) OR (MH "mental health+") OR (MH "Depression+") OR (MH "Anxiety+") OR (MH "Suicide+") OR (MH "Burnout, Professional+")) **AND** ((TI Stigma* OR AB Stigma*) OR (TI attribute* OR AB attribute*) OR (TI perception* OR AB perception*) OR (TI attitude* OR AB attitude*) OR (MH "Stigma+") OR (MH "perception+")) OR (MH "Attitude+") **AND** ((TI educational intervention* OR AB educational intervention*) OR (TI program* OR AB program*) OR (TI training* OR AB training*) OR (TI course* OR AB course*) OR (TI curriculum* OR AB curriculum*)) |
| PsycINFO | (doctor*.ti,ab OR physician*.ti,ab OR "medical practitioner*".ti,ab OR "healthcare professional*".ti,ab OR "student doctor*".ti,ab OR "medical student*".ti,ab OR "medical trainee*".ti,ab OR "med student*".ti,ab OR "general practitioner*".ti,ab) **AND** ("mental health".ti,ab OR "mental illness*".ti,ab OR "mental well-being".ti,ab OR "mental wellbeing".ti,ab OR "psychosocial health".ti,ab OR "psychosocial well-being".ti,ab OR "psychosocial wellbeing".ti,ab OR depression.ti,ab OR anxiety.ti,ab OR suicide.ti,ab OR burnout.ti,ab) **AND** (Stigma*.ti,ab OR attribute*.ti,ab OR perception*.ti,ab OR attitude*.ti,ab) **AND** (educational intervention*.ti,ab OR program*.ti,ab OR training*.ti,ab OR course*.ti,ab OR curriculum*.ti,ab) |
